# Antimicrobial resistance and characterisation of staphylococci isolated from healthy Labrador retrievers in the United Kingdom

**DOI:** 10.1186/1746-6148-10-17

**Published:** 2014-01-14

**Authors:** Vanessa M Schmidt, Nicola J Williams, Gina Pinchbeck, Caroline E Corless, Stephen Shaw, Neil McEwan, Susan Dawson, Tim Nuttall

**Affiliations:** 1 Department of Infection Biology, The University of Liverpool, Leahurst Campus, Neston, UK; 2 Department of Epidemiology and Population Health, The University of Liverpool, Leahurst Campus, Neston, UK; 3 Infection and Immunity, Royal Liverpool University Hospital, Liverpool, UK; 4 UK VetDerm, Coalville, UK; 5 The Royal (Dick) School of Veterinary Studies, Easter Bush Campus, University of Edinburgh, Midlothian, UK; 6 The University of Liverpool School of Veterinary Science, Leahurst Campus, Chester High Road, Neston, Wirral CH64 7TE, UK

**Keywords:** Coagulase-postive staphylococci, Coagulase-negative staphylococci, Meticillin-resistant, Dogs, MALDI-TOF-MS, *Tuf* gene, *Nuc* gene, Antimicrobial-susceptibility

## Abstract

**Background:**

Coagulase-positive (CoPS) and coagulase-negative (CoNS) staphylococci are normal commensals of the skin and mucosa, but are also opportunist pathogens. Meticillin-resistant (MR) and multidrug-resistant (MDR) isolates are increasing in human and veterinary healthcare. Healthy humans and other animals harbour a variety of staphylococci, including MR-CoPS and MR-CoNS. The main aims of the study were to characterise the population and antimicrobial resistance profiles of staphylococci from healthy non-vet visiting and non-antimicrobial treated Labrador retrievers in the UK.

**Results:**

Nasal and perineal samples were collected from 73 Labrador retrievers; staphylococci isolated and identified using phenotypic and biochemical methods. They were also confirmed by matrix-assisted laser desorption ionisation time-of-flight mass spectrometry (MALDI-TOF-MS), PCR of the *nuc* gene and PCR and sequencing of the *tuf* gene. Disc diffusion and minimum inhibitory concentration (MIC) susceptibility tests were determined for a range of antimicrobials. In total, 102 CoPS (*S. pseudintermedius* n = 91, *S. aureus* n = 11) and 334 CoNS isolates were detected from 99% of dogs in this study. In 52% of dogs CoNS only were detected, with both CoNS and CoPS detected in 43% dogs and CoPS only detected in 4% of dogs. Antimicrobial resistance was not common among CoPS, but at least one MDR-CoNS isolate was detected in 34% of dogs. MR-CoNS were detected from 42% of dogs but no MR-CoPS were isolated. *S. epidermidis* (52% of dogs) was the most common CoNS found followed by *S. warneri* (30%) and *S. equorum* (27%), with another 15 CoNS species isolated from ≤ 15% of dogs. *S. pseudintermedius* and *S. aureus* were detected in 44% and 8% of dogs respectively.

**Conclusions:**

MR- and MDR-CoPS were rare. However a high prevalence of MR- and MDR-CoNS were found in these dogs, even though they had no prior antimicrobial treatment or admission to veterinary premises. These findings are of concern due to the potential for opportunistic infections, zoonotic transmission and transmission of antimicrobial resistant determinants from these bacteria to coagulase positive staphylococci.

## Background

Staphylococci are normal commensal bacteria of the skin and mucous membranes of humans and other animals. They can be differentiated by their ability to produce coagulase, with coagulase positive (CoPS) staphylococci regarded as more pathogenic than coagulase negative (CoNS) species [1-5].

Healthy humans and other animals may harbour multiple species and strains of staphylococci. *Staphylococcus aureus* is the main human commensal CoPS species and is carried in the nasal cavity of approximately 30% of healthy people [6]. *S. epidermidis* is the most common CoNS isolated from the nares, perineum, inguinal skin, axillae and interdigital skin in man [2,7]. The main commensal CoPS of dogs, *S. pseudintermedius*[8], has been isolated from 37% to 92% of healthy dogs [9-14], while *S. aureus* is carried by 4.3% to 12% of healthy dogs [10,12,15-20]. Other species isolated from the mucosa and skin of healthy dogs include the CoPS *S. schleiferi* subspecies *coagulans*[10,21] and numerous CoNS (*S. schleiferi* subspecies *schleiferi*, *S. epidermidis*, *S. haemolyticus*, *S. saprophyticus*, *S. devriesei*, *S. warneri, S. simulans, S. xylosus, S.capitus, S. caprae,* and *S. sciuri*) [12,15,22-26]. The carriage rate of CoNS isolated from the nasal mucosae of healthy dogs was reported to be 38% in one large cross-sectional study [15].

Staphylococci are frequent opportunistic pathogens and commensal isolates are the most common source of infection in humans [3] and dogs [12,16,27]. Antimicrobial resistance can increase the morbidity, mortality and treatment cost of staphylococcal infections. Meticillin (oxacillin) resistance associated with carriage of the *mec*A gene confers resistance to all β-lactam antimicrobials [28]. The *mec*A gene is located on a large mobile genetic element, the staphylococcal cassette chromosome *mec* (SCC*mec*), enabling horizontal transmission between staphylococcal isolates [29]. Meticillin resistant staphylococci (MRS) are important pathogens in human and veterinary healthcare and are often multi-drug resistant (MDR; resistant to three or more classes of antimicrobial) [30-35], extremely limiting therapeutic options. MRSP clones with a broader resistance spectrum than MRSA or MR-CoNS are increasingly reported in domestic animals throughout Europe, USA and Canada [32,34]. MR-CoNS are associated with infections in humans and animals [31,36-38]. In humans the most prevalent species is MR *S. epidermidis* (MRSE), which may be a reservoir of MR for *S. aureus*[39,40]*.* In addition, the SCC*mec* cassette of the major European MRSP clone (ST71-J-t02-II–III) [34] consists of a combination of SCC*mec* II from MRSE and SCC*mec* III from MRSA [41].

The prevalence of MRSA and MRSP carriage in healthy humans and dogs in the community is low [11,18,36,42-47]. However, human community-based surveys report a wider range of carriage rates for MR-CoNS (11–50%) [39,48,49]. MR-CoNS have also been isolated from the carriage sites of 13% of healthy dogs [23,50]. The reported prevalence of MRS is higher in animals exposed to veterinary healthcare environments and antimicrobial therapy [47,51-53] suggesting that these are risk factors for colonisation.

Previous studies looking at the commensal staphylococci in dogs have concentrated on CoPS species, particularly MR-CoPS species, the CoNS group or MR-CoNS species [9-11,13,14,17,23,50], but no study has characterised the complete canine commensal staphylococcal population. Moreover, reporting of the antimicrobial treatment history of dogs in these studies have been inconsistent. The aim of this study was to characterise the mucosal staphylococcal population structure and antimicrobial resistance profiles in healthy Labrador retrievers in the UK in the absence of antimicrobial pressure. This will be important in understanding changes in staphylococcal populations and their antimicrobial susceptibility patterns in dogs exposed to antimicrobials and other risk factors.

## Methods

### Study population

Labrador retriever dogs were recruited for the study from dog shows in the UK between November 2010 and June 2011. One healthy dog was enrolled from each household if the dog had not received topical or systemic antimicrobial therapy, or had not been admitted to a veterinary clinic within the last 12 months. All dog owners gave written informed consent before enrolment in this study and completed a questionnaire regarding potential risk factors for the carriage of antimicrobial resistant bacteria. The University of Liverpool School of Veterinary Science ethics committee approved the study protocol.

### Staphylococci

#### Specimen collection and bacterial isolation

One nasal swab and one perineal swab were collected from each dog (Copan Eswab LQ Amies Minitip Nylon Flocked Applicator, Appleton Woods, Birmingham, UK). A sterile swab was either inserted 5 mm into one nostril or rubbed on the skin of the perineum for 3–5 seconds before being placed in Amies transport media, stored at 4°C and processed within 36 hours. Swabs were incubated aerobically overnight at 37°C in nutrient broth with 6.5% sodium chloride. The broth was streaked onto mannitol salt agar (MSA), oxacillin resistance screening agar (ORSA) supplemented with 2 μg/ml of oxacillin and Columbia 5% horse blood agar (CAB), and incubated aerobically overnight at 37°C. Where present, isolates typical of staphylococci were selected from all plates, sub-cultured onto CAB and incubated aerobically overnight at 37°C. Fresh staphylococcal cultures on CAB were subject to Gram stain (Sigma-Aldrich Company Ltd., Gillingham, UK), tested for catalase (Sigma-Aldrich Company Ltd., Gillingham, UK) and free coagulase production (Rabbit plasma, Pro-Lab, Bromborough, UK) according the manufacturer’s instructions and stored at − 80°C in Microbank vials (Pro-Lab, Bromborough, UK). All media were obtained from LabM Ltd, Bury, UK.

#### Antimicrobial susceptibility testing

Disc diffusion testing was performed on all staphylococcal isolates in accordance with the Clinical and Laboratory Standards Institute (CLSI) and the following panel of ten antimicrobial discs were applied: 1 μg oxacillin (OX), 1 μg ciprofloxacin (CIP), 10 μg gentamicin (GM), 10 μg fusidic acid (FA), 30 μg cefalexin (CFX), 30 μg cefovecin (CVN), 25 μg trimethoprim-sulfamethoxazole (TS), 10 μg tetracycline (Tet), 2 μg clindamycin (CD) and 5 μg vancomycin (Va) [54]. All the discs were purchased from MAST Group Ltd., Liverpool, UK, except for CVN, which were obtained from Oxoid, Basingstoke, UK. Micro-dilution susceptibility testing (Trek Diagnostic Systems, Cleveland, Ohio, USA) was performed on a subset of the CoNS isolates using the same antimicrobial panel, except vancomycin [54]. Interpretation was based on the CLSI guidelines for animal species-specific zone diameter (mm) interpretive standards and minimal inhibitory concentration (MIC; mg/l) breakpoints for veterinary pathogens or human-derived interpretive standards when available. The European Committee on Antimicrobial Susceptibility Testing (EUCAST) zone diameter interpretive standards and MIC breakpoints were used for CIP and FA [55]. The breakpoints used for interpretation of OX resistance were a zone of inhibition of ≤ 17 mm and MIC ≥ 0.5 mg/l for S*. pseudintermedius* and CoNS, and ≤ 10 mm and MIC ≥ 4 mg/l for *S. aureus*[56,57]. The breakpoints used for interpretation of resistance to CVN as a zone of inhibition of ≤ 19 mm and MIC ≥ 8 mg/l in accordance with the manufacturer’s recommendations. The reference strain *S. aureus* ATCC®25923 (LGC Standards, Teddington, UK) was used for quality control for MIC and zone diameter determinations.

#### DNA extraction

Three colonies of each staphylococcal isolate were homogenised in 90 μl of sterile distilled water (SDW) and 10 μl of lysostaphin (1 mg/ml; Sigma-Aldrich Company Ltd., Gillingham, UK) and vortexed for 5 seconds. The suspensions were then incubated at 37°C for 10 minutes and heated at 100°C for 10 minutes before adding 400 μl of SDW. Samples were stored at 4°C.

#### Characterisation of antimicrobial resistance genes

PCR assays were performed to detect the presence of *mec*A gene (Table 1) in staphylococcal isolates that were phenotypically resistant to oxacillin. All the PCR assays were performed with 0.5 μl of each primer (10 pmol/μl), 1 μl of DNA and 1.1x PCR master mix (ReddyMix™, Thermo Fisher Scientific Inc., Surrey, UK) made up to a total reaction volume of 25 μl. Molecular grade water (Sigma-Aldrich Company Ltd., Gillingham, UK) was used as the negative control in all PCR assays. PCR products were analysed by agarose gel (1.5%) electrophoresis and the DNA fragments were visualised under UV light after ethidium bromide staining.

**Table 1:** **Details of PCR assays used in this study for *nuc*, *tuf *** **and *mec*** **A gene identification**

**Primer**	**Sequence (5′-3′)**	**Amplicon size (bp)**	**Annealing Temperature (°C)**	**Control strain**	**Reference**
au-F3	TCGCTTGCTATGATTGTGG	359	57	*S. aureus* ATCC®25923 (LGC Standards, Teddington, UK)	[77]
au*-*nucR^*^	GCCAATGTTCTACCATAGC				
pse-F2	TRGGCAGTAGGATTCGTTAA	926	57	*S. pseudintermedius* (clinical isolate)	
pse-R5^*^	CTTTTGTGCTYCMTTTTGG				
SSnucF	AATGGCTACAATGATAATCACTAA	526	57	*S. schleiferi* subspecies *coagulans* ATCC®49545	
SS*nuc*R^*^	CATATCTGTCTTTCGGCGCG				
*tuf-*F	GCCAGTTGAGGACGTATTCT	412	55	*S. epidermidis* ATCC*®*12228	[105]
*tuf-*R	CCATTTCAGTACCTTCTGGTAA				
*mec*AF	TGGCTATCGTGTCACAATCG	310	55	MRSA (clinical isolate)	[103,104]
*mec*AR	CTGGAACTTGTTGAGCAGAG				
mA1	TGCTATCCACCCTCAAACAGG	286	57		
mA2	AACGTTGTAACCACCCCAAGA				

(*multiplex assay).

### Species identification

#### Genotypic species identification

PCR assays to detect the presence of the *nuc* genes of *S. pseudintermedius, S. aureus* and *S. schleiferi* were performed on all CoPS isolates using Qiagen® Multiplex PCR Mix (Qiagen, Crawley, UK), according to the manufacturer’s instructions with minor modifications. In short, the PCR assays were performed in a reaction volume of 25 μl, consisting of 5 μl of bacterial DNA extract, 12.5 μl of master mix, 2.5 μl of 10x primer mix (2 μM of each primer) and 5 μl of RNase-free water. The cycling conditions consisted of an initial activation step at 95°C for 15 minutes, followed by 30 cycles of 95°C for 30 seconds, 57°C for 90 seconds and 72°C for 60 seconds, and a final extension step at 72°C for 10 minutes (Table 1).

#### MALDI-TOF-MS

All isolates were subjected to matrix-assisted laser desorption ionisation time-of-flight mass spectrometry (MALDI-TOF-MS) according to the manufacturer’s protocol. Raw spectra were analysed by the MALDI Biotyper 2.0 software programme with default settings (Bruker Daltonics, Bremen, Germany). The extraction method was performed as previously described [58] on overnight colonies grown on CAB at 37°C and all isolates were tested in duplicate. The bacterial test standard (*E. coli* DH5 alpha, Bruker, Bremen, Germany) was used for calibration before each experiment and included in duplicate on each target plate. The mass peak profiles were matched to the reference database and a score generated based on similarity [59].

#### Sequencing

Two subsets of isolates detected from our group of dogs underwent sequencing following PCR amplification of the *tuf* gene [59,60]; a control group of CoNS isolates (n = 27) identified by MALDI-TOF-MS and a test group of isolates (n = 52) that had not been identified by MALDI-TOF-MS. Initial PCR assays were performed using HotStarTaq® Master Mix Kit (Qiagen, Crawley, UK) in a 25 μl reaction volume with an initial activation step at 95°C for 15 minutes followed by 35 cycles of 95°C for 30 seconds, 55°C for 30 seconds and 72°C for 30 seconds, with a final extension step of 72°C for 5 minutes according to the manufacturer’s protocol. The resulting amplicons were sequenced using BigDye Terminator version 1.1 cycle sequencing (Applied Biosystems, Foster City, CA, USA) according to the manufacturer’s protocol on the ABI3131 genetic analyser at the Department of Microbiology, Royal Liverpool University Hospital. The sequences were aligned using the ABI Sequencing analysis software, with contiguous sequences matched to the GenBank database using the Basic Local Alignment Search Tool (BLAST) [61] and positively identified if there was ≥ 98% sequence similarity with a reference sequence. *S. epidermidis* ATCC*®*12228 was used as the control strain.

### Statistical analysis

Data were analysed using SPSS software package (SPSS 20.0 for Mac, SPSS Inc, Chicago, Illinois).

To examine the association between isolation of *S. pseudintermedius* with each of the 16 different CoNS species Pearson’s chi-square was calculated (*P* < 0.003; Bonferroni correction). To examine the association between MR and MDR with potential risk factors (previous antimicrobial therapy or hospitalisation within 12 months of enrolment, health-care or large animal-association by in-contact people or pets) identified from the questionnaires Pearson’s chi-square was calculated (*P* < 0.0125; Bonferroni correction). To examine the agreement between antimicrobial susceptibility tests by disc diffusion and MIC a *kappa* statistic was calculated [62] and an independent *t*-test was conducted to compare the MIC of oxacillin resistant CoNS isolates that were either positive or negative for the *mec*A gene.

## Results

### Staphylococci

#### Specimen collection

Seventy-three Labrador retriever dogs were recruited. Twenty-one dogs were aged between 3 to 12 months, 25 dogs were aged between 12 months to 2 years, and 27 dogs were > 2 years old, with 35 female dogs and 38 male dogs in total.

#### Bacterial isolation

Staphylococci were isolated from in 72 out of 73 dogs (99%; 95% confidence interval (CI): 99.6-95.8) and from both sample sites in the majority of dogs (78%; 95% CI: 67.3, 86.0). Isolation of staphylococci from the nasal mucosae (16%, 95% CI 9.7, 26.6) or perineum (4%, 95% CI 1.4, 11.4) only occurred in a small number of dogs. If only the nasal mucosae had been sampled, CoPS (all *S. pseudintermedius*) would not have been detected in seven dogs (10%, 95% CI 4.7, 18.5) and CoNS in six dogs (8%, 95% CI 3.8, 16.8). CoNS were detected in the majority of dogs (95%, 95% CI 86.7, 97.8) either alone (52%, 95% CI 40.8, 63.1) or with CoPS (43%, 95% CI 31.8, 53.9). Detection of CoPS alone was significantly less common (4%, 95% CI 1.4, 11.4). In total, there were 436 staphylococcal isolates; 102 of which were CoPS and 334 were CoNS isolates.

#### Antimicrobial susceptibility testing by disc diffusion

**Figure 1:**
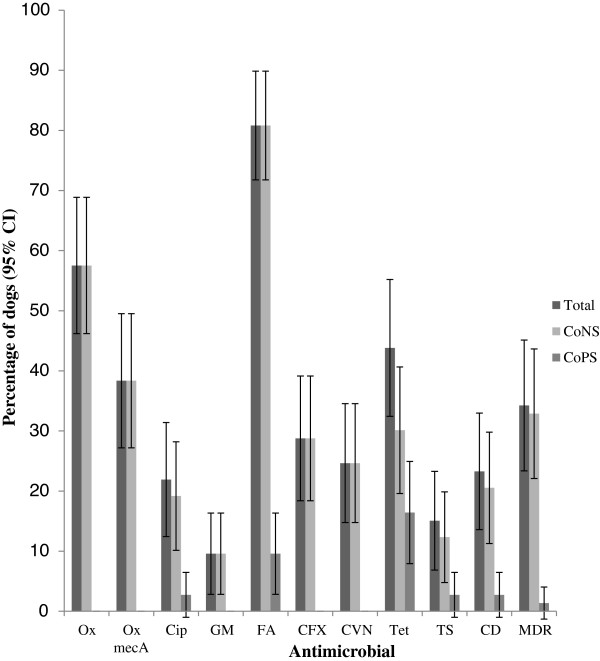
**The proportion of dogs (n = 73) carrying at least one staphylococcal isolate with resistance to each antimicrobial, MDR and *mec*A gene positive oxacillin resistance by the disc diffusion method.** Total = CoNS and CoPS.

The overall prevalence of antimicrobial resistance among the isolates detected in this study appeared high, with at least one MDR isolate detected in 34% of dogs. Antimicrobial resistant CoNS isolates were detected in more dogs than antimicrobial resistant CoPS isolates for OX, GM, FA, CFX, CVN and CD and MDR (Figure 1). At least one OX resistant isolate was detected in 58% dogs (n = 126 oxacillin resistant isolates), but resistance to the other tested β-lactam antimicrobials, CVN (25%) and CFX (29%), was less common. Few CoPS demonstrated antimicrobial resistance; isolates from twelve dogs had Tet resistance (all *S. pseudintermedius*), seven with FA resistance *(S. pseudintermedius* = 5, *S. aureus* = 3); two with TS resistance (both *S. pseudintermedius*); two with CD resistance (*S. pseudintermedius* = 1*, S. aureus* = 1) and two with CIP resistance (both *S. pseudintermedius*). MDR CoPS was detected from only one dog (*S. pseudintermedius* with FA, Tet and CD resistance) (Figure 1).

#### MIC compared to disc diffusion testing for antimicrobial resistance

Micro-dilution susceptibility testing (Trek Diagnostic Systems, Cleveland, Ohio, USA) was performed on 172 CoNS isolates, of which 52 were OX susceptible and 120 were OX resistant by disc diffusion. The OX resistant isolates were further divided into those found to be positive (n = 74) or negative (n = 46) for carriage of the *mec*A gene by PCR. The strength of agreement between antimicrobial resistance detected by MIC and disc diffusion was very good for OX, GM, CVN, Tet and CD resistance, good for CFX and TS resistance and moderate for CIP (*Kappa* = 0.593) and FC resistance (*Kappa* = 0.589). MIC testing identified more isolates as resistant to OX, GM, CFX, CVN and Tet compared to disc diffusion, and disc diffusion identified more isolates as resistant to CIP, FA, TS and CD compared to MIC testing (Table 2).

**Table 2:** Cross tabulation of the results of 172 staphylococcal isolates classified as resistant or susceptible to the antimicrobials tested in this study by both MIC and disc diffusion testing

**Antimicrobial resistance**			**MIC**		
			No	Yes	Total
Oxacillin (OX)	**Disc diffusion**	No	50	10	60
		Yes	2	110	112
		Total	52	120	172
		*Kappa* = 0.842			
			No	Yes	Total
Oxacillin *mec*A positive		No	115	0	115
		Yes	1	56	57
		Total	116	56	172
		*Kappa* = 0.987			
			No	Yes	Total
Ciprofloxacin (CIP)		No	146	0	157
		Yes	14	12	15
		Total	160	12	172
		*Kappa* = 0.593			
			No	Yes	Total
Gentamicin (GM)		No	156	1	157
		Yes	0	15	15
		Total	156	16	172
		*Kappa* = 0.965			
			No	Yes	Total
Fusidic acid (FA)		No	36	5	41
		Yes	25	106	131
		Total	61	111	172
		*Kappa* = 0.589			
			No	Yes	Total
Cefalexin (CFX)		No	117	15	132
		Yes	0	40	40
		Total	117	55	172
		*Kappa* = 0.784			
			No	Yes	Total
Cefovecin (CVN)		No	130	11	141
		Yes	0	31	31
		Total	130	42	172
		*Kappa* = 0.810			
			No	Yes	Total
Tetracycline (T10)		No	148	1	149
		Yes	0	23	23
		Total	148	24	172
		*Kappa* = 0.975			
				Yes	Total
Trimethoprim-sulfamethoxazole (TS)		No	156	2	158
		Yes	3	11	14
		Total	159	13	172
		*Kappa* = 0.799			
			No	Yes	Total
Clindamycin (CD)		No	148	2	150
		Yes	4	18	22
		Total	152	20	172
		*Kappa* = 0.837			

#### Characterisation of antimicrobial resistance genes

**Figure 2:**
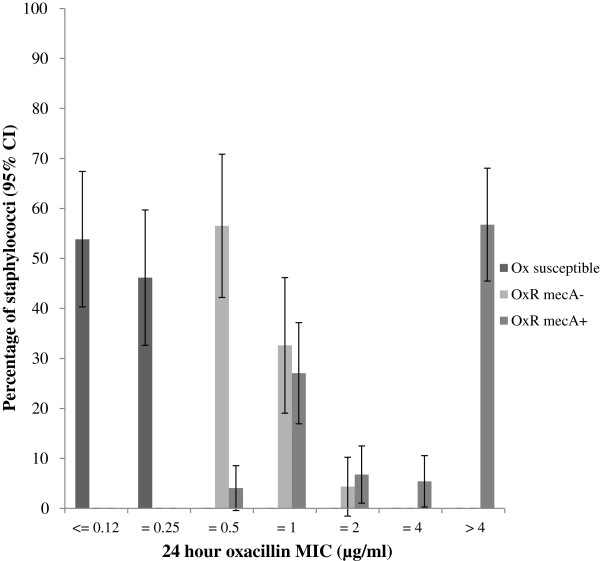
**The MIC (μg/ml) data for staphylococcal isolates (n = 172).** The isolates consisted of 52 oxacillin susceptible isolates, 46 oxacillin resistant *mec*A negative isolates and 74 oxacillin resistant *mec*A positive isolates.

**Figure 3:**
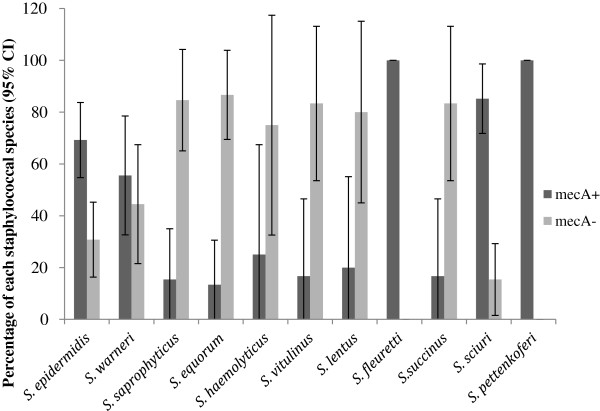
**The percentage of each oxacillin-resistant staphylococcal species by disc diffusion and MIC that was either positive (*mec*** **A+) or negative (*mec*** **A-) for the *mec*** **A gene.**

Of the 126 OX resistant CoNS isolates detected by disc diffusion, 75 isolates (60%, 95% CI 51, 68) from 31 dogs (42%, 95% CI 32, 54) were positive for the *mec*A gene (Figure 1). Nine additional oxacillin resistant isolates were detected by MIC and two of these were positive for the *mec*A gene, resulting in two additional dogs with MR-CoNS and one additional dog with phenotypic oxacillin resistant CoNS. There was a significant difference between the MIC of *mec*A positive (M = 3.84, SD = 0.18) and *mec*A negative isolates (M = 0.97, SD = 0.12, *P* < 0.001). In addition the epidemiological breakpoint for OX resistant CoNS isolates with *mec*A gene carriage isolated in this study was consistent with the clinical CLSI breakpoint (≥ 0.5 mg/l) (Figure 2). Eleven different CoNS species (*S. epidermidis, S. warneri, S. sciuri, S. equorum, S. fleurettii, S. vitulinus, S. saprophyticus, S. haemolyticus, S. lentus, S. succinus* and *S. pettenkoferi*) were found to carry the *mec*A gene. Among oxacillin resistant CoNS species, *S epidermidis* and *S. sciuri* were more likely to carry the *mec*A gene than *S. saprophyticus*, *S. equorum*, *S. vitulinus* and *S. succinus* (Figure 3)*.* MRSE isolates were detected in 18 dogs (25%, 95% CI 14.8, 34.5), meticillin-resistant *S. warneri* were detected in 7 dogs (10%, 95% CI 2.8, 16.3) and meticillin-resistant *S. sciuri* were detected in 5 dogs (7%, 95% CI 1.1, 12.6). The remaining species were only isolated from one or two dogs. MDR *mec*A positive CoNS were detected in 19 dogs (26%, 95% CI 17.3, 37.1). There was no significant association between detection of MR-CoNS or MDR isolates and potential risk factors tested in this study (Pearson’s chi-square; *P* < 0.0125).

#### Species identification

Phenotypic and biochemical methods identified 436 isolates as *Staphylococcus* species. Using a combination of *nuc* gene PCR, MALDI-TOF-MS and sequencing of the *tuf* gene, 399 isolates (92%, 95% CI 88.5, 93.8) were identified to the species level. MALDI-TOF-MS identified 345 isolates to the species level including 264 of 334 CoNS isolates (79%, 95% CI 74.4, 83.1). Amplification and sequencing of the *tuf* gene identified 33 out of 51 CoNS isolates (65%, 95% CI 51, 76.4) to the species level (n = 11 species; ≥ 98% sequence similarity) and an additional control group (n = 27) of CoNS isolates that had also been identified by MALDI-TOF-MS. There was 100% agreement between the two methods for the identification of the control group. In particular, sequencing of the *tuf* gene identified all of the *S. fleurettii*, *S. arlettae* and *S. pettenkoferi* isolates, 12 isolates closely related to *S. felis* (96% sequence similarity) and an additional 15 isolates to the genus level (*Staphylococcus* spp. ≥ 98% sequence similarity). PCR amplification of the *nuc* gene detected all of the *S. aureus* n = 11 (100%, 95% CI 74.1, 100) and *S. pseudintermedius* isolates n = 91 (100%, 95% CI 96.0, 100). There was 100% agreement of this assay with MALDI-TOF-MS for the identification of *S. aureus* isolates, however MALDI-TOF-MS only identified 69 out of 91 *S. pseudintermedius* isolates.

**Figure 4:**
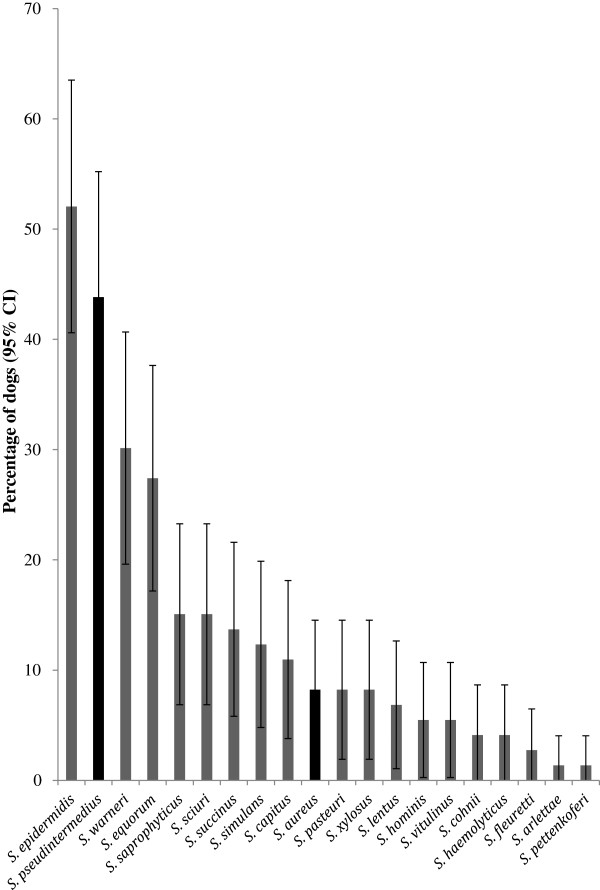
**The percentage of dogs (n = 73) carrying each staphylococcal species identified in this study by MALDI-TOF-MS, PCR of the *nuc *** **gene and sequencing of the *tuf *** **gene (CoNS grey and CoPS black).**

Overall from the combined results using PCR amplification of the *nuc* gene, MALDI-TOF-MS and sequencing of the *tuf* gene we detected *S. epidermidis* in 52% (95% CI 41, 63) and *S. pseudintermedius* in 44% (95% CI 33, 55) of the dogs*. S. warneri* and *S. equorum* were the next most common species, isolated from 30% and 27% of dogs respectively, and the remaining staphylococcal species were carried by no more than 15% of the dogs. *S. aureus* was detected in 6 of the dogs, exclusively from the nasal mucosae, and usually with *S. pseudintermedius* (88%, 95% CI 52.9, 97.8). *S. pseudintermedius* was concurrently isolated with 16 different CoNS species, although there was no significant association between the presence of *S. pseudintermedius* and any CoNS species (Pearson’s chi-square; *P* < 0.003) (Figure 4 and Table 3).

**Table 3:** **The number of staphylococcal isolates identified to species level by MALDI-TOF-MS, *nuc *** **gene PCR (CoPS), and *tuf *** **gene sequencing**

**Staphylococcal species**	**Number of isolates**	**Number (%) of positive dogs**	**Number (%) identified by MALDI-TOF-MS**	**Number (%) of CoPS identified by *nuc *** **PCR**	**Number (%) identified by *tuf *** **gene sequencing**
*S. pseudintermedius*	91	32 (44)	70 (77)	91 (100)	0
*S. aureus*	11	6 (8)	11 (100)	11 (100)	0
*S. epidermidis*	67	38 (52)	64 (96)	N/A	3 (4)
*S. warneri*	35	22 (30)	35 (100)	N/A	0
*S. equorum*	39	20 (27)	36 (92)	N/A	3 (8)
*S. saprophyticus*	19	11 (15)	15 (79)	N/A	4 (21)
*S. sciuri*	27	11 (15)	21 (78)	N/A	6 (22)
*S. succinus*	19	10 (14)	16 (84)	N/A	3 (16)
*S. simulans*	15	9 (12)	15 (100)	N/A	0
*S. capitus*	7	8 (11)	7 (100)	N/A	0
*S. pasteuri*	8	6 (8)	8 (100)	N/A	0
*S. xylosus*	17	6 (8)	17 (100)	N/A	0
*S. lentus*	9	5 (7)	4 (44)	N/A	5 (56)
*S. hominis*	6	4 (5)	6 (100)	N/A	0
*S. cohnii*	5	3 (4)	3 (60)	N/A	2 (40)
*S. vitulinus*	14	3 (4)	13 (93)	N/A	1 (7)
*S. haemolyticus*	4	3 (4)	4 (100)	N/A	0
*S. fleurettii*	*4*	2 (3)	0	N/A	4 (100)
*S. arlettae*	1	1 (1)	0	N/A	1 (100)
*S. pettenkoferi*	1	1 (1)	0	N/A	1 (100)
Total ID	399^d^	72^e^	345^f^	101^f^	33^f^
*Staphylococcus* spp.	3	N/A	N/A	N/A	3
Species related to *S. felis*	12	N/A	N/A	N/A	12
No ID	22	N/A	N/A	2	3
Total	436^a^	73^b^	436^c^	102^c^	51^c^

Values in the table are expressed as total numbers and percentage in parenthesis where applicable. ^a^Total number of isolates in study, ^b^total number of dogs in study, ^c^total number of isolates tested by each method, ^d^total number of isolates with positive identification (ID), ^e^number of dogs with staphylococcal detection, ^f^number of isolates with positive ID from each method.

## Discussion

This is the first study incorporating MALDI-TOF-MS to successfully characterise commensal staphylococcal populations in a group of healthy dogs in the absence of antimicrobial pressure. We isolated staphylococci from 99% of our dogs, with 95% carrying CoNS and 47% carrying CoPS. The relative prevalence of the staphylococci concurs with other published studies in humans [2,3], horses [63-67] and dogs [15,17,68], although the overall staphylococcal prevalence was double that reported for healthy vet visiting dogs [15]. This could be related to the study population and techniques, as we sampled both the nose and the perineum to increase detection of CoPS [12,13,68,69].

We were able to assign 92% of the staphylococcal isolates to 20 different species, including 18 CoNS. This is the first study to demonstrate such diversity in dogs, and carriage of this number of different species has only been previously reported for humans [2,10,12,15,21-26,70]. The most common species was *S. epidermidis*, which was detected in 52% of the dogs, mainly from the nasal cavity. This is similar to human reports [71], but apart from one canine study [23], *S. epidermidis* has not been commonly reported in different animal species [67,72,73]. *S. pseudintermedius* was the second most common species and the most common CoPS detected, also in agreement with previous reports [9-11,13]. Unlike *S. epidermidis*, *S. pseudintermedius* was carried equally in the nose and on the perineum, suggesting that this species may have a wider range of mucosal niches. Very few dogs carried *S. aureus* (8%), which is comparable to other studies that reported carriage rates of approximately 7% from healthy vet visiting dogs [12,15]. The majority of the CoNS in our study were human-associated and included *S. epidermidis*, *S. hominis, S. haemolyticus, S. capitus, S. saprophyticus, S. warneri, S. cohnii, S. simulans, S. pettenkoferi and S. pasteuri.* Human associated CoNS species have previously been isolated from dogs, horses, cows and pigs [23,67,72,74-76]. The other CoNS species isolated from our dogs are reported as indigenous to animals (*S. equorum*, *S. vitulinus, S. arlettae S. sciuri*, *S. lentus* and *S. fleurettii*) [2].

We used several methods to identify staphylococcal isolates to species level. Multiplex PCR for the *nuc* gene is an accurate, rapid and cost efficient method to speciate CoPS [77], which identified 100% of our *S. pseudintermedius* (n = 91) and 100% of our *S. aureus* isolates (n = 11). Recently MALDI-TOF-MS has been reported as a rapid and reliable method to characterise CoNS, *S. aureus* and *S. intermedius* group (SIG) strains [59,72,78-80]. MALDI-TOF-MS identified all of our *S. aureus* isolates, 77% of our *S. pseudintermedius* isolates and 79% of our CoNS isolates, identified by phenotypic and biochemical characteristics, to the species level. Similar results for the identification of *S. aureus*, *S. pseudintermedius* and CoNS by MALDI-TOF-MS, in comparison to molecular methods, have been reported [79-81]. The overall performance of MALDI-TOF-MS to speciate the staphylococcal isolates in this study, similar to other reports [80], is likely to be directly related to the database, which at the time of analysis consisted mainly of common human-derived species and only one *S. pseudintermedius* strain. However species level identification will improve as more highly characterised reference isolates are added to the database. Amplification and sequencing of the *tuf* gene is regarded as the gold standard to speciate CoNS isolates [59,60]. This method identified 77% of the tested staphylococcal isolates (n = 79) to the species level. The performance of this method in our study may have been affected by the lack of certain-animal derived isolates representing different species in the database. Additionally, we may have improved identification by sequencing a larger region of the *tuf* gene. We sequenced a previously described 412 base pair region of the *tuf* gene that was reported to have successfully identified 88% of human-derived staphylococcal strains [60]. However, a more recent publication that sequenced a 660 bp region of the *tuf* gene, reported 98.9% identification of 186 human and animal-derived staphylococcal strains.

We did not detect any MR-CoPS isolates. Other studies of healthy dogs have similarly reported a low prevalence [15,82,83]. In contrast, 58% of the dogs in our study carried at least one CoNS isolate with phenotypic meticillin resistance and 42% carried a meticillin resistant *mec*A positive isolate. Other studies have also reported high levels of meticillin resistance among CoNS isolates from humans [31,35,84], horses [23,64-66] and livestock [72,85]. However, the prevalence of MR-CoNS carriage in our study is markedly higher than the levels reported in other community canine studies [15,23,50,74,83]. High community carriage rates of MR-CoNS are of concern for animals and humans, as these organisms may not only be reservoirs of resistance genes for CoPS [39,40,86], but also act as pathogens [31,36-38,87-89]. Cross-transmission is reported to be an important mechanism for dissemination of MRS [49,90], and transmission between dogs and in-contact humans may occur in the community and in veterinary premises [36,83].

Nine different CoNS species carried the *mec*A gene in our study with MRSE detected in 25% of our dogs. MRSE is the predominant MR-CoNS species in humans both in hospital and community settings [39,48,49], and has been reported in one study investigating nasal carriage of MRS in dogs [23]. Other canine studies have isolated meticillin resistant *S. sciuri* and meticillin resistant *S. warneri*[23,74]. Our research found that the majority of the *S. sciuri* and *S. fleurettii* isolates were *mec*A positive, which is consistent with other studies in humans, livestock and horses [35,64,66,67,72].

MDR CoNS (n = 38) were isolated from 34% of dogs in this study. MDR was generally associated with resistance to β-lactams, FA and additional antimicrobials. In particular MDR-MRSE were resistant to at least four antimicrobial classes tested in our study. A similar finding was reported in a study of hospitalised animals, medical equipment and veterinary staff [68]. MDR among CoNS isolates is widely reported [15,49,72,73,91] and may be associated with the carriage of multiple antimicrobial resistance genes on SCC*mec* cassettes [40]. In contrast, the majority of our commensal CoPS isolates were susceptible to a broad range of antimicrobials (apart from Tet), in line with previous reports for clinical isolates [92-94] and isolates from healthy vet-visiting dogs [15]. There was good to very good agreement between disc and MIC antimicrobial susceptibility testing apart for FC and CIP. These two antimicrobials were the only ones where human breakpoints were applied and emphasises potential species differences in pharmacokinetic and pharmacodynamic data for individual antimicrobials.

The *mec*A gene was not identified in 40% of the phenotypic oxacillin resistant isolates in this study and may include some isolate duplication due to our sampling methods. Other studies have reported phenotypic meticillin resistance with absence of the *mec*A gene in staphylococci [95-98]. Our OX-resistant *mec*A negative isolates may be truly negative for the *mec*A gene as they were less likely to be resistant to the other antimicrobials tested in this study, including CVN and CFX, and had significantly lower MICs compared to the OX resistant *mec*A positive isolates. It is possible that they had low-level resistance associated with other mechanisms such as hyperproduction of β-lactamases [99], or production of an oxacillin-specific β-lactamases [100]. There are bovine mastitis CoNS isolates with oxacillin MICs of 0.5 – 1 mg/l that lack the *mec*A gene [97], and the CLSI guidelines state that ‘oxacillin interpretive criteria may overcall resistance for these CoNS strains’ [57]. In addition, many of the published PCR assays to identify and characterise the *mec*A gene have been developed for MRSA [101-104] and may therefore lack sensitivity for some CoNS isolates. However, other authors have successfully employed the same methods for *mec*A detection among CoNS isolates as used in our study [68,98,105]. Nevertheless it is possible that additional PCR assay [106], or latex agglutination for PBP2a [107] may have improved the sensitivity of *mec*A detection or detected phenotypic *mec*A-associated resistance in our oxacillin resistant *mec*A negative isolates.

Our study had some limitations, including the small sample size. Still, these dogs yielded 436 staphylococcal isolates and a high prevalence of resistance was identified among the CoNS isolates even in the absence of antimicrobial exposure. Another weakness was that the study population was limited to one breed (Labrador retrievers) and the dogs were recruited at dog shows. Kennelled dogs have been shown to have higher levels of antimicrobial resistance in faecal *E. coli* than individually owned and non-kennelled dogs [108]. Kennelling was transient in our dogs, but this may have affected the results. Many of the dogs came from multi-dog households but only one dog from each household was sampled to avoid cluster effects.

## Conclusions

This is the first comprehensive study of commensal staphylococcal populations in a group of healthy dogs. Staphylococci, particularly CoNS, form a normal part of the canine commensal population and were detected from almost all the dogs. The most commonly isolated staphylococcal species in this group of dogs was *S. epidermidis*, although a wide variety of other human- and animal-associated CoNS were found. CoPS were less common, and the major species was *S. pseudintermedius*. Antimicrobial resistance among the CoPS was uncommon, and no MRSP or MRSA were isolated, however the sample size was small. Antimicrobial resistance (including MDR and meticillin resistance) was common among the CoNS isolates, even though this was a community population of healthy dogs in the absence of direct-antimicrobial pressure or veterinary contact. The clinical significance of commensal CoNS and MR-CoNS is unclear, but *S. epidermidis* carries a number of virulence factors and is an increasing cause of nosocomial and community-acquired infections in humans. The possibility of similar infections escalating in companion animals cannot be excluded. In addition, there is potential for cross-species transmission of antimicrobial resistant bacteria and exchange of resistance determinants between bacterial species. In particular, MR- and MDR-CoNS may provide a reservoir of antimicrobial resistance genes that could rapidly spread within bacterial populations under the selection pressure exerted by antimicrobial therapy. Further longitudinal studies in healthy dogs and in dogs receiving antimicrobials are required to assess the population diversity, antimicrobial resistance profiles and persistence of antimicrobial resistant staphylococci in dogs.

## Abbreviations

CoPS: Coagulase positive staphylococci; CoNS: Coagulase negative staphylococci; MDR: Multidrug resistance; MR: Meticillin resistance; MIC: Minimum inhibitory concentration; MRSA: Meticillin resistant *Staphylococcus aureus*; MRSP: Meticillin resistant *staphylococcus pseudintermedius*; MRSE: Meticillin resistant *staphylococcus epidermidis*; OX: Oxacillin; CIP: Ciprofloxacin; CFX: Cefalexin; CVN: Cefovecin; TS: Cotrimazole; Tet: Tetracycline; CD: Clindamycin.

## Competing interests

Vanessa Schmidt, Neil McEwan, Stephen Shaw and Tim Nuttall have received other unrelated funding from Zoetis (previously Pfizer Animal Health UK). The authors declare that there are no financial or non-financial competing interests.

## Authors’ contributions

VS was responsible for sample collection and processing, data analysis and writing the manuscript. NJW was responsible for advising on the microbiology methodology used in the study and interpretation of results and contributed to the writing of the manuscript. GP advised on statistical analysis. NM advised on ethical permission and sample collection and contributed to the writing of the manuscript. SS assisted sample collection and contributed to the writing of the manuscript. CEC was responsible for advising on and performing sequencing. SD advised on interpretation of results and contributed to the writing of the manuscript. TN advised on sample collection, interpretation of results and contributed to the writing of the manuscript. NW, GP, NM, SD and TN supervised VS during this project. All authors were involved in the design of this project and reviewed and approved the final manuscript.
